# Examining Whether Onfield Motor Incoordination Is Associated With Worse Performance on the SCAT5 and Slower Clinical Recovery Following Concussion

**DOI:** 10.3389/fneur.2020.620872

**Published:** 2021-03-01

**Authors:** Grant L. Iverson, Ryan Van Patten, Andrew J. Gardner

**Affiliations:** ^1^Department of Physical Medicine and Rehabilitation, Harvard Medical School, Boston, MA, United States; ^2^Department of Physical Medicine and Rehabilitation, Spaulding Rehabilitation Hospital, Charlestown, MA, United States; ^3^Spaulding Research Institute, Charlestown, MA, United States; ^4^Sports Concussion Program, MassGeneral Hospital for Children, Boston, MA, United States; ^5^Home Base, A Red Sox Foundation and Massachusetts General Hospital Program, Charlestown, MA, United States; ^6^Hunter New England Local Health District Sports Concussion Program, Newcastle, NSW, Australia; ^7^Priority Research for Stroke and Brain Injury, School of Medicine and Public Health, The University of Newcastle, Callaghan, NSW, Australia

**Keywords:** rugby league, video analysis, brain injury, clinical assessment, return to play

## Abstract

**Objective:** To examine the relationship between video-identified onfield motor incoordination, the acute assessment of concussion, and recovery time during three seasons of National Rugby League (NRL) play.

**Methods:** Blows to the head (“head impact events”) were recorded by sideline video operators and medical staff. Any player with a suspected concussion underwent a Head Injury Assessment in which he was taken off the field and medically evaluated, including the administration of the Sports Concussion Assessment Tool, 5th Edition (SCAT5). Video footage was later examined to determine the presence or absence of onfield motor incoordination following the head impact event.

**Results:** Motor incoordination was identified in 100/1,706 head impact events (5.9%); 65 of the 100 instances of motor incoordination (65.0%) were ultimately medically diagnosed with a concussion. In 646 athletes for whom SCAT5 data were available, those with motor incoordination were more likely to report both dizziness and balance problems than those without motor incoordination, but there were no group differences on an objective balance test. Additionally, there was no relationship between presence/absence of motor incoordination and number of games missed or time to medical clearance for match play.

**Conclusion:** In NRL players, motor incoordination is a readily observable onfield sign that is strongly associated with a medical diagnosis of concussion and with self-reported dizziness/balance problems. However, onfield motor incoordination is not associated with objective balance performance and it is not predictive of time to recover following concussion.

## Introduction

Motor incoordination following a sport-related concussion is visualized as difficulty standing and walking. Examples include falling when attempting to stand, stumbling gait, walking at an angle, and falling after either standing or beginning to walk. Because motor incoordination often can be plainly seen on the field, from the sideline, from the stands, and on television, it is a useful visual sign of a possible concussive injury.

Many professional sporting leagues such as the National Football League (1, 2), National Hockey League (2), Australian Football League (2), Cricket Australia (2), professional rugby union (3), and the National Rugby League (NRL) (2) have implemented video surveillance to help with the identification of concussions (2, 4), with trained spotters in the arena, and with spotters having access to multiple camera angles and replay capabilities (in a centralized location). Motor incoordination is included in the international consensus definition of video signs of concussion (4).

The NRL is the elite professional club rugby league competition in Australia. In one study, a single observer watched 210 games on video from the 2014 National Rugby League (NRL) season and coded possible video signs of concussion associated with 127,062 tackles (5). Athletes were slow to get up 2,240 times. However, motor incoordination was observed only 102 times. Of those with observed motor incoordination, more than half were medically diagnosed as having sustained a concussion.

Over the past several years, the NRL has refined the process for identifying concussions by implementing a rule that allows for medical staff to evaluate a player who sustains an in-game blow to the head to determine whether or not he might have sustained a concussion (5, 6). The league also has a sideline injury surveillance system with personnel examining video footage, in real time, to identify blows to the head. The purpose of this study is to determine whether those with acute motor incoordination on video show evidence of having a worse concussion from a clinical perspective. We hypothesized that those athletes who show this onfield sign would be more likely to be medically diagnosed with a concussion than those who did not show this onfield sign. We also hypothesized that those athletes who showed motor incoordination on the field would be more likely to report dizziness and balance difficulties on the sideline, and they would perform worse on an objective balance test on the sideline, compared to those who did not show this video sign. We retained the null hypothesis regarding whether motor incoordination would be associated with recovery time following concussion because we had no prior studies or a theory to justify a directional hypothesis.

## Methods

### Participants

All players who participated in any game during three NRL seasons (2017–2019) were included in this study. The total number of games played each year for the entire league was 201 (192 regular season and 9 post season). Head impact events identified by either the video sideline injury surveillance system or the team doctor were recorded and uploaded to the *GamePlan* application. As part of their collective bargaining agreement, all athletes consented *a priori* to have their deidentified injury data used in research endorsed by the Rugby League Research Committee. This study was approved by the Institutional Human Ethics Committee at the University of Newcastle (Ref No. H-2012-0344).

### Sideline Injury Surveillance

During all NRL matches, personnel examine video footage in real-time in the stadium, and identify and record blows to the head or body that might cause injury (termed a “head impact event”). These sideline operators manage the live video feed and provide technical support for requests from team medical staff to review incidents via replay. The operators tag all head impact events and all match play injuries (concussions and other injuries) in the video surveillance system. The total number of head impact events include blows to the head identified by the operators, medical staff, or both.

### Head Injury Assessment

A Head Injury Assessment in the NRL is initiated if there is an observed impact to a player's head and the medical staff have concern about a possible concussion. The NRL Head Injury Assessment allows the team medical staff to remove and medically evaluate an athlete from match play. There is a 15-min time period for the assessment, during which the club doctor administers the Sports Concussion Assessment Tool Fifth Edition (SCAT5) (7) using a tablet-based web hosted CSx data platform (6, 8).

### SCAT5

The SCAT5 (7) has been promoted by the Concussion in Sport Group as a standardized acute clinical assessment for athletes suspected of concussion (9–11). Previous versions, the SCAT (9), SCAT2 (12), and SCAT3 (13) have been published over the past 15 years, and the SCAT is widely used in professional and amateur sports (14–18). The SCAT5 includes eight individual sections: (i) potential signs of concussion include classic definitional criteria of traumatic brain injury (e.g., loss of consciousness and amnesia); (ii) the Glasgow Coma Scale is recorded in case of neurological deterioration and to document more severe brain injury, if present; (iii) sport-specific orientation and amnesia assessed using the five Maddocks questions; (iv) demographic variables, self-reported concussion history, potential outcome modifiers, and medications; (v) a concussion symptom evaluation consisting of 22 symptoms that are graded on a dimensional scale from 0 (none) to 6 (severe); (vi) a cognitive assessment based on the standardized assessment of concussion (SAC) (19) which assesses time orientation, immediate memory, concentration, and delayed recall components; (vii) a neurological screening evaluation involving a cervical/neck examination, eye movements, finger-to-nose test, and tandem gait; and (viii) a measure of postural stability using the modified version of the Balance Error Scoring System (mBESS) (20).

### Video Analysis

Video footage of head impact events were clipped and loaded on the *GamePlan* Application. The senior author has been provided access to the NRL's *GamePlan* Application subscription and all video clips by the NRL. Access to the video footage of head impact events was typically available within an hour of the event, and often sooner. Most clips provided multiple camera angles of the event, in normal speed, and in slow motion. The video clips ranged in duration from ~1–5 min. The senior author reviewed all video clips on an Apple iPhone and coded all variables, including game play characteristics, tackle characteristics, and player characteristics, as previously described (21), in addition to coding video signs of potential concussion. Motor incoordination for this study was defined by the international consensus definition of video signs of concussion (4, 22), as: “Appears unsteady on feet (including losing balance, staggering/stumbling, struggling to get up, falling) or in the upper limbs (including fumbling). May occur in rising from the playing surface or in the motion of walking/running/skating” (page 1265).

### Statistical Analysis

For continuous variables, descriptive statistics include the mean (SD), median, and range (interquartile range [IQR]), while categorical variables are presented as number included (*n*) and/or percentage of the total. Relationships between categorical variables were tested using chi-square tests. The Mann Whitney *U*-test examined differences in number of days to be medically cleared to return to full contact or match play because this variable was not normally distributed. The Mann Whitney *U* was also used to test for group difference in SCAT5 variables due to non-normality. SCAT5 analyses excluded SAC Immediate Memory, Delayed Memory, and Total Score data from players who were administered the 10-word list because the majority of the sample completed the 5-word list. Effect sizes are presented as correlation coefficients (23, 24) for Mann Whitney *U*-tests and as odds ratios for chi-square tests. Statistical significance was considered at an alpha level of 0.05 and all analyses were performed in IBM SPSS (IBM Corp. Released 2017. IBM SPSS Statistics V.27).

## Results

**Figure 1:**
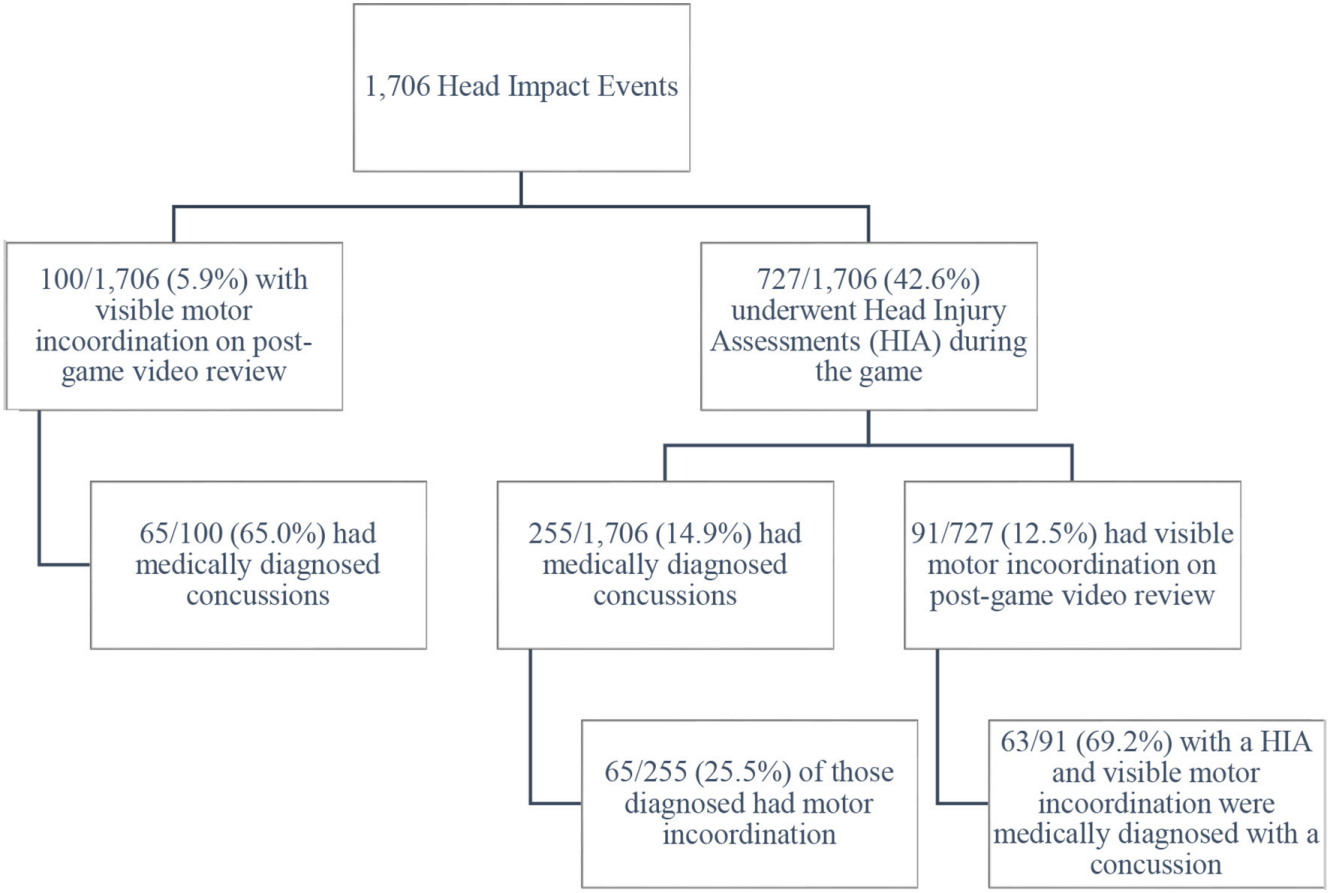
Onfield motor incoordination following head impacts. Nine Head Impact Events were not followed by Head Injury Assessments but were ultimately associated with medically diagnosed concussions. This explains the two different denominators in the bottom corners of the flow diagram (100 and 91).

There were 1,706 head impact events, of which 727 (42.6%) led to clinical assessments and 255 (14.9%) led to medically diagnosed concussions. The motor incoordination sign was identified in 100 of 1,706 head impact events (5.9%) and 91 of 727 clinical assessments (12.5%). Of the 91 cases of motor incoordination who underwent clinical assessments, 63 (69.2%) were medically diagnosed with a concussion. Of the total 100 instances of onfield motor incoordination observed on video, 65 (65.0%) were ultimately associated with a concussion diagnosed by medical personnel. Of the 255 players with a medical diagnosis of concussion, 65 (25.5%) had video evidence of motor incoordination (see Figure 1). Those athletes undergoing a clinical evaluation who had video-identified motor incoordination were more likely to be diagnosed with a concussion than those without video-identified incoordination [χ^2^ (1, *n* = 712) = 69.7, *p* ≤ 0.001, odd ratio (OR) = 6.5, 95% confidence interval (CI) = 4.0–10.5].

With respect to the medical diagnosis of concussion (i.e., the outcome) and onfield motor incoordination, there were 65 true positives, 190 false negatives, 35 false positives, and 1,516 true negatives. This resulted in sensitivity of 0.25, specificity of 0.98, positive predictive value of 0.65, negative predictive value of 0.89, positive likelihood ratio of 11.30, and negative likelihood ratio of 0.76 for this onfield sign.

### Motor Incoordination and SCAT5 Scores: Total Sample

SCAT5 data were available from 646 athletes. SCAT5 component scores are presented by group (presence/absence of motor incoordination) in Table 1. Participants with motor incoordination endorsed more SCAT5 symptoms and performed worse on Maddocks questions, SAC total scores, delayed recall, and orientation than those without motor incoordination. There were no group differences in immediate memory, concentration, or mBESS total errors. A subset of the sample (124/646, 19.2%) endorsed dizziness and a subset (59/646, 9.1%) endorsed balance problems. Compared to those without video-identified motor incoordination, those athletes with this sign were more likely to report both dizziness [χ^2^ (1, *n* = 646) = 11.9, *p* = 0.001, OR = 2.4, 95% CI = 1.4–3.9] and balance problems [χ^2^ (1, *n* = 646) = 15.3, *p* < 0.001, OR = 3.2, 95% CI = 1.7–5.9] on the sideline.

**Table 1:** Full sample SCAT5 component scores by presence vs. absence of motor incoordination.

	**Motor incoordination**						**No motor incoordination**									
**Test Component**	***n***	**Mean**	**SD**	**Mdn**	**IQR**	**Range**	***n***	**Mean**	**SD**	**Mdn**	**IQR**	**Range**	**U**	**Z**	***p***	***r***
Symptom severity	89	7.3	14.2	1.0	0–7	0–83	557	4.0	10.0	0.0	0–3	0–126	29265.5	2.9	0.004	0.11
Number of symptoms	89	3.4	4.8	1.0	0–5	0–20	557	1.9	3.6	0.0	0–2	0–21	29898.0	3.3	0.001	0.13
Maddocks questions total	89	4.5	1.2	5.0	5–5	0–5	557	4.8	0.8	5.0	5–5	0–5	21550.5	4.0	0.000	0.16
SAC total score	86	25.2	6.1	27.5	23–29	1–30	527	27.1	3.8	28.0	26–29	0–30	19590.5	−2.0	0.041	−0.08
Orientation	89	4.1	1.5	5.0	4–5	0–5	557	4.6	0.8	5.0	4–5	0–5	19766.0	−3.9	<0.001	0.15
Immediate memory	86	13.9	2.7	15.0	14–15	0–15	545	14.2	21.9	15.0	15–15	0–15	22657.0	−0.6	0.53	−0.02
Concentration	89	3.6	1.5	4.0	3–5	0.5	557	4.0	1.1	4.0	3–5	0–5	22568.0	−1.4	0.15	0.06
Delayed recall	86	3.7	1.8	4.0	3–5	0–8	527	4.2	1.2	5.0	4–5	0–7	19280.5	−2.4	0.02	−0.10
mBESS total errors	89	4.2	3.2	3.0	2–6	0–16	557	3.7	3.0	3.0	1–5	0–20	26539.5	1.1	0.28	0.04

*Effect sizes for group differences are presented as correlation coefficients, with the following interpretations (23, 24): 0.1 = small, 0.3 = moderate, 0.5 = large. SAC, Standardized Assessment of Concussion; mBESS, Modified Balance Error Scoring System. The sample sizes for Immediate Memory, Delayed Recall, and the SAC Total Score are smaller because the athletes who completed the 10–item word list were excluded*.

### Motor Incoordination and SCAT5 Scores: Subgroup With Diagnosed Concussions

There were 212 players who were medically diagnosed with concussions for whom SCAT5 results were available. The SCAT5 component scores are presented by group (presence/absence of motor incoordination) in Table 2. The only significant group difference occurred in SAC orientation, where those with motor incoordination performed worse than those without motor incoordination. All other comparisons were non-significant.

**Table 2:** Concussed athletes' SCAT5 component scores by presence vs. absence of motor incoordination.

	**Motor incoordination**						**No motor incoordination**									
**Test component**	***n***	**Mean**	**SD**	**Mdn**	**IQR**	**Range**	***n***	**Mean**	**SD**	**Mdn**	**IQR**	**Range**	**U**	**Z**	**p**	**r**
Symptom severity	63	10.0	16.1	4.0	0–14	0–83	157	9.5	12.7	4.0	1–13.5	0–66	4664.5	−0.7	0.51	0.05
Number of symptoms	63	4.6	5.2	3.0	0–7	0–20	157	4.7	5.1	3.0	1–7.5	0–21	4740.5	−0.5	0.63	0.03
Maddocks questions total	63	4.3	1.4	5.0	4–5	0–5	157	4.6	0.9	5.0	5–5	0–5	4396.5	−1.8	0.07	0.12
SAC total score	60	23.8	6.7	26.5	22–29	1–30	147	25.5	3.9	26.0	24–28	5–30	4100.5	−0.8	0.43	−0.06
Orientation	63	3.8	1.6	4.0	3–5	0–5	157	4.4	1.0	5.0	4–5	1–5	4132.5	−2.1	0.03	0.14
Immediate memory	60	13.5	3.1	15.0	14–15	0–15	152	13.9	2.1	15.0	14–15	3–15	4543.5	−0.5	0.96	−0.03
Concentration	63	3.3	1.6	4.0	2–5	0–5	157	3.6	1.3	4.0	3–5	0–5	4631.0	−0.8	0.45	0.05
Delayed recall	60	3.2	1.8	4.0	2–5	0–8	147	3.5	1.5	4.0	3–5	0–5	4150.5	−0.7	0.49	−0.05
mBESS total errors	63	4.7	3.4	3.0	2–7	0–16	157	5.2	3.9	5.0	2–7	0–20	4606.0	−0.8	0.42	0.05

*Athletes included in this table were diagnosed with a concussion by their team medical staff. Effect sizes for group differences are presented as correlation coefficients, with the following interpretation (23, 24): 0.1 = small, 0.3 = moderate, 0.5 = large. SAC, Standardized Assessment of Concussion; mBESS, Modified Balance Error Scoring System. The sample sizes for Immediate Memory, Delayed Recall, and the SAC Total Score are smaller because the athletes who completed the 10-item word list were excluded*.

### Time to Medical Clearance to Return to Sports

For the 255 medically diagnosed concussions, data on athletes' games missed were available for 218 (85.5%). Due to non-normality in the number of games missed for each participant, the variable was transformed into a binary state (0 games vs. 1+ games). There was no association between games missed and presence or absence of motor incoordination [χ^2^ (1, *n* = 218) = 0.47, *p* = 0.49, OR = 1.3, 95% CI = 0.6–2.9].

Of the 255 medically diagnosed concussions, data on time to medical clearance for match play (in days) were available for 219 (85.9%). The Mann-Whitney *U*-test examining differences in days to medical clearance by presence (mdn = 6.0, range = 0–79) and absence (mdn = 6.0, range = 0–79) of motor incoordination was not significant, *U* = 5477.0, *z* = 1.6, *p* = 0.12, *r* = 0.11.

## Discussion

Motor incoordination following a concussion is visualized as falling when attempting to stand, stumbling gait, walking at an angle, or falling after standing or beginning to walk. The sign was not visualized very often in the current study, occurring in ~6% of the 1,706 documented blows to the head during three seasons of professional rugby league matches. When it was present, however, the athlete was likely to have sustained a concussion. In the present study, the sign was observed 100 times over the course of three seasons, and in 2 out of 3 cases, the player was subsequently diagnosed with a concussion. As hypothesized, those athletes undergoing a clinical evaluation on the sideline who had video-identified motor incoordination were significantly more likely to be diagnosed with a concussion than those who did not show this sign. Moreover, as hypothesized, those with motor incoordination were more likely to endorse balance problems and dizziness on the sideline (i.e., as self-reported symptoms). However, they did not perform worse than those without motor incoordination on an objective measure of balance (the mBESS), which was inconsistent with our hypothesis. Finally, those athletes who showed motor incoordination on the field did not have worse outcomes following concussion. They were medically cleared to return to sports in a similar number of days and they missed a similar number of games compared to those who did not show this sign.

It seems unusual that those with visible onfield motor incoordination did not perform significantly worse on the mBESS in the total sample or in the subsample that was medically diagnosed with concussion. They were significantly more likely to self-report dizziness and balance difficulties, although most did not report these symptoms. Although the mBESS is commonly used to measure static balance and postural stability following concussion, it is a brief screening measure with moderate reliability and sensitivity (25–27). Given that the mBESS is conducted on a firm surface, whereas the original BESS also includes the same balance tests conducted on a foam surface, the mBESS might not be sufficiently refined or sensitive to detect subtle changes in balance and postural stability, even minutes after a concussion, in some cases.

One quarter of concussed NRL players in our study were determined to have motor incoordination, and this rate is consistent with a recent report in National Football League players, where motor incoordination was present in 28% of the concussed athletes (28), but lower than the rate of this video sign reported in a prior study of NRL players (5). NRL players with observed onfield motor incoordination in the current study were more likely to be medically diagnosed with concussions than those who sustained blows to the head but did not show motor incoordination. The relationship between onfield motor incoordination and concussion diagnosis has been documented previously. Partially consistent with current study results, onfield motor incoordination has high specificity, low sensitivity, and mixed findings with respect to positive and negative predictive value in the prediction of sports-related concussions in professional athletes (28–31).

### Limitations

This study as several methodological limitations. First, the 1,706 videos were viewed and coded by a single rater (AJG), consistent with some of our prior studies (5, 22, 32). The senior author leads a research program relating to the video analysis of concussion in rugby league (5, 6, 8, 21, 22, 32–35), and also has worked on the sideline in support of the medical staff at rugby league games, and thus has extensive experience with both the sport and the coding. Nonetheless, results could differ, modestly, if another person coded the videos and consensus was achieved. Second, each team has its own medical staff and there could be some differences in how they identify and diagnose concussions. The medical staff receive continuing education relating to concussion diagnosis and management that might reduce some of the variability. Third, and relatedly, SCAT5 interrater reliability is imperfect and could benefit from further investigation, so it is possible that slightly different results would have been obtained with different SCAT5 examiners. Fourth, a substantial percentage of NRL players self-identify their race and ethnicity as Pacifika or Indigenous Australia, and little is known about whether these subgroups of players perform differently on the SCAT5. Fifth, our video analysis was conducted *post-hoc*, without in-game time pressure, and so our findings cannot be assumed to generalize to real-time video analysis. Sixth, our measurement of clinical recovery from concussion was limited to games missed and medical clearance for match play. A more detailed examination of clinical recovery may have produced different results. Seventh, the majority of the NRL teams used the 5-word rather than the 10-word list on the SAC, so we only analyzed data from the 5-word list. If teams had used the 10-word list, we may have detected additional group differences (motor incoordination vs. no motor incoordination) due to the enhanced difficulty of the task. Finally, and importantly, there could be a degree of both circularity and confirmatory bias that enhanced the strength of the association between video evidence of motor incoordination and a medical diagnosis of concussion. That is, if the medical staff observes a blow to the head, followed by a player demonstrating motor incoordination upon standing, that sets up a higher index of suspicion, or even an expectation, for concussion. This could influence how the physician interprets the examination findings, possibly lowering the threshold for diagnosing a concussion.

### Conclusions

Motor incoordination is uncommon following a blow to the head in the NRL. However, when present, it is strongly associated with a medical diagnosis of concussion—similar to findings from other studies in professional sports (28–31). However, onfield motor incoordination was not predictive of slower time to recover from concussion in this study.

## Data Availability Statement

The statistical analyses and data supporting the conclusions of this article will be made available by the authors to qualified researchers for research purposes, without undue reservation.

## Ethics Statement

This study was reviewed and approved by the Institutional Human Ethics Committee at the University of Newcastle.

## Author Contributions

GI conceptualized the study, assisted with the literature review, conceptualized the statistical analyses, helped conduct the statistical analyses, drafted sections of the manuscript, edited the manuscript, and approved the final manuscript. RVP assisted with reviewing the literature, helped conduct the statistical analyses, drafted portions of the manuscript, edited the manuscript, and approved the final manuscript. AG served as the principal investigator, secured regulatory ethics for the study, helped conceptualize the study, examined and coded all of the videos used in the study, created the database, edited the manuscript, and approved the final manuscript. All authors contributed to the article and approved the submitted version.

## Conflict of Interest

AG serves as a scientific advisor for hitIQ, Ltd. He has a clinical practice in neuropsychology involving individuals who have sustained sport-related concussion (including current and former athletes). He has been a contracted concussion consultant to Rugby Australia since July 2016. He has received travel funding or been reimbursed by professional sporting bodies, and commercial organizations for discussing or presenting sport-related concussion research at meetings, scientific conferences, workshops, and symposiums. Previous grant funding includes the NSW Sporting Injuries Committee, the Brain Foundation (Australia), an Australian-American Fulbright Commission Postdoctoral Award, a Hunter New England Local Health District, Research, Innovation and Partnerships Health Research & Translation and Clinical Research Fellowship Scheme, and the Hunter Medical Research Institute (HMRI), supported by Jennie Thomas, and the HMRI, supported by Anne Greaves. GI has a clinical and consulting practice in forensic neuropsychology, including expert testimony, involving individuals who have sustained mild TBIs (including athletes). He has received research funding from several test publishing companies, including ImPACT Applications, Inc., CNS Vital Signs, and Psychological Assessment Resources (PAR, Inc.). He has received research support from the Harvard Integrated Program to Protect and Improve the Health of NFLPA Members, and grant support from the National Football League. He serves as a scientific advisor for Sway Operations, LLC, Highmark, Inc., and for NanoDX® (formerly BioDirection, Inc.). The remaining author declares that the research was conducted in the absence of any commercial or financial relationships that could be construed as a potential conflict of interest.
